# Disparate Roles of Cell–Cell Contact and Cytokine Secretion in an In Vitro Model of the Seminoma Microenvironment

**DOI:** 10.3390/ijms26136173

**Published:** 2025-06-26

**Authors:** Patrick Fruth, Juliane Luft, Lucas Klaus, Tobias J. Legler, Holger M. Reichardt, Fabian A. Gayer

**Affiliations:** 1 Institute for Cellular and Molecular Immunology, University Medical Center Göttingen, 37073 Göttingen, Germany; patrick.fruth@stud.uni-goettingen.de (P.F.); juliane.luft@stud.uni-goettingen.de (J.L.); lucas.klaus1@med.uni-goettingen.de (L.K.); fabian.gayer@med.uni-goettingen.de (F.A.G.); 2 Department of Transfusion Medicine, University Medical Center Göttingen, 37075 Göttingen, Germany; tlegler@med.uni-goettingen.de; 3 Clinic of Urology, University Medical Center Göttingen, 37075 Göttingen, Germany

**Keywords:** TCam-2, seminoma, tumor microenvironment, T cells, monocytes

## Abstract

Type II testicular germ cell tumors (TGCTs) are the most common solid malignancies in young men and are classified into seminomas and non-seminomatous subtypes. Seminomas are known for their highly pro-inflammatory tumor microenvironment (TME) with abundant immune cell infiltration. While previous work has demonstrated that the seminoma-derived cell line TCam-2 induces immune cell activation in co-culture and undergoes phenotypic changes itself, the underlying mechanisms remained unclear. To explore the role of direct cell–cell interaction and the effects mediated by soluble mediators such as cytokines, we conducted co-culture experiments of TCam-2 cells with purified human T cells or monocytes, including Transwell assays and treatments with IL-6, TNFα, or their respective blocking antibodies Tocilizumab and Adalimumab. In this way, we found that immune cell activation, indicated by enhanced secretion of pro-inflammatory cytokines and an upregulation of activation markers, strongly depended on direct physical contact between both cell types. Nonetheless, we also unveiled the role of soluble mediators in both immune cell activation and promoting a shift in TCam-2 cells from a seminoma-like phenotype to a more dedifferentiated phenotype, suggesting that cytokines critically shape the TME. These observations highlight the complexity of tumor–immune interactions in the seminoma microenvironment, offering new insight into immune-driven dynamics in TGCTs.

## 1. Introduction

Testicular germ cell tumors (TGCTs) are a clinical success story in terms of curability, mainly due to the extraordinary efficiency of platin-based polychemotherapy [1]. Nonetheless, they still present major challenges related to epidemiology and the complexity of the tumor microenvironment (TME). Recent advances in molecular profiling, the characterization of tumor–immune interactions, and systems biology are gradually reshaping our understanding of TGCTs. Hence, current research aimed at integrating these insights into clinical practice holds great promise for more targeted and less toxic management of this tumor entity.

Despite the overall low incidence of TGCTs of approximately 1% of all male cancers, they are still the leading solid malignancy in young men aged 18 to 35 [2]. They present with a rising incidence, notable geographic disparity, and considerable association with environmental and genetic risk factors [3]. TGCTs are derived from primordial germ cells and can be classified into three separate types [4]. The most frequent one, namely type II tumors, arises due to a precursor lesion known as germ cell neoplasia in situ (GCNIS) and can be subdivided into seminomas and non-seminomatous germ cell tumors, characterized by distinct histological and genetic features and a roughly similar frequency distribution [5,6].

The TME is a complex network composed of tumor cells, stromal components, various immune cell subtypes, and soluble factors that critically influence tumor progression and therapeutic response. As a matter of fact, immune cells within the TME can exert anti-tumoral and pro-tumoral functions, depending on their activation state and the prevailing signals [7,8]. In contrast to most other tumor entities, TGCTs and, in particular, seminomas are characterized by a pronounced pro-inflammatory TME [9,10,11,12]. The stimulation of immune cells in the TME can occur via two fundamentally distinct mechanisms: direct cell–cell interaction or signaling through soluble mediators. Direct interactions involve physical contact between immune and tumor cells, mediated by membrane-bound ligands and their respective receptors, including stimulatory and inhibitory signaling molecules like CD28, CD80, PD-1, and PD-L1 [13]. This contact is crucial for shaping the transcriptome and secretome of immune cells and thus their functional activities in the TME. In contrast, soluble mediators, such as the pro-inflammatory cytokines IL-6 and TNFα, various chemokines, and growth factors, orchestrate immune activation in a paracrine or autocrine manner. In this way, they shape the inflammatory milieu, guide immune cell recruitment, modulate activation thresholds, and influence proliferation, survival, and cellular identity [14,15]. It is noteworthy that recent insights into the cellular interactions in the TME have further unveiled a communication network established between tumor cells, cancer-associated fibroblasts, and macrophages, which relies on both soluble factors and cell–cell contact [16].

T cells and macrophages account for the largest proportion of infiltrating immune cells within the TME of TGCTs [10,11,17]. This pro-inflammatory characteristic stands in sharp contrast to most other tumor entities, which instead promote an anti-inflammatory and thus pro-tumoral TME. Interestingly, testicular cancer cells seem to grow and differentiate in a way that is dependent on the composition of the TME. That being said, previous work has demonstrated a shift in seminomatous tumor cells towards a more dedifferentiated phenotype under inflammatory conditions in vitro, characterized by an upregulation of pluripotency markers such as *SOX2* and a diminished expression of seminoma-specific markers like *SOX17* [18]. Importantly, such dedifferentiation is associated with more aggressive tumor behavior, which highlights its clinical relevance [19]. Interestingly, the polarization of infiltrating immune cells has also been observed to impact clinical parameters, confirming once again that the TME shapes the quality of the immune response in TGCT patients in a relevant manner [17,20,21].

The immune system and its influence on prognosis and therapy for cancer have become more and more important in daily medicine over the last few decades. Therefore, understanding the balance and interplay of cell–cell interactions between tumor and immune cells, as well as the effects mediated by soluble mediators, is essential for further unraveling the dynamics in TGCTs. It can be expected that insights gained from this knowledge will become instrumental in developing new therapeutic approaches based on the blockade of checkpoint molecules and the provision or neutralization of cytokines.

In vitro models provide a reproducible platform for studying cellular mechanisms within the TME. The use of defined cell lines and isolated immune cell subtypes allows us to analyze cell-specific effects on T cell and monocyte behavior and tumor cell responses in the absence of disturbing systemic influences present in vivo. The TCam-2 cell line is well characterized and established as a reliable model of seminomas [22,23,24,25]. Previous work has shown the pronounced pro-inflammatory response of immune cells in co-cultures with TCam-2 cells in vitro, characterized by a strong release of cytokines and a significant upregulation of markers indicating immune cell activation. Additionally, TCam-2 cells showed an altered gene expression profile, particularly for markers related to stemness and differentiation like *OCT3/4*, *NANOG*, *SOX2,* and *SOX17*, following co-culture with immune cells [18,22,25,26].

IL-6 and TNFα, two central pro-inflammatory players in cytokine networks, influence processes including carcinogenesis, metabolism, proliferation, and angiogenesis in the TME [27]. Furthermore, it has been shown that IL-6 protects tumor cells from therapy-induced DNA damage [28]. In TGCTs, a varying expression profile of IL-6 was observed depending on its localization in the TME, and it was also found to differ between metastatic and non-metastatic seminomas [29]. In the context of excessive activation of the immune system, inhibitors of TNFα and IL-6 signaling pathways, including Adalimumab and Tocilizumab, are an integral part of state-of-the-art therapeutic approaches to treating autoimmune diseases and cancer [30]. While Adalimumab is a neutralizing antibody directed against soluble TNFα, Tocilizumab binds to the membrane-bound IL-6 receptor, thereby preventing cytokine signaling in target cells.

The presence of communication between infiltrating immune cells and tumors cells in the TME of TGCTs is beyond any doubt, but its specific impact on tumor progression and differentiation is mostly unclear. While previous studies have shown that TCam-2 cells induce immune cell activation in an in vitro co-culture model of the TME, the relative contribution of direct cell–cell contact as opposed to soluble mediators such as cytokines remains unclear. This study thus aims to explore these mechanisms to better understand the TME of seminomas. To this end, we performed co-culture experiments of primary human T cells and monocytes with TCam-2 cells using Transwell inserts to address the relevance of physical cell–cell contact. To investigate the impact of cytokines locally produced in the TME of TGCTs, we used the monoclonal antibodies Adalimumab and Tocilizumab, conditioned medium, and recombinant human IL-6 and TNFα. In this way, we unveiled differing contributions from direct tumor–immune interactions and soluble mediators in shaping the pro-inflammatory TME and the phenotype of testicular tumor cells in seminomas.

## 2. Results

### 2.1. Role of Cell–Cell Contact in TCam-2 Co-Cultures with Immune Cells

Previous work has unveiled that co-cultures of TCam-2 cells with freshly isolated T cells or monocytes show features reminiscent of the pro-inflammatory TME in seminomas, particularly the immune cells’ activated phenotype [18]. However, it remained unclear if TCam-2 cells stimulate T cells and monocytes via direct cell–cell contact or the secretion of soluble mediators.

**Figure 1:**
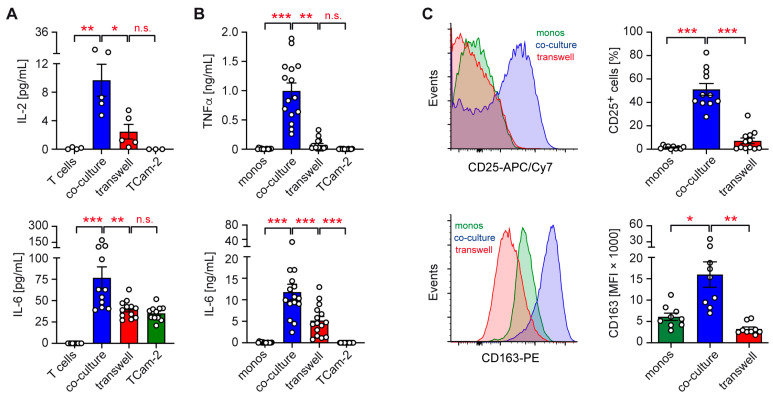
Cytokine secretion and surface marker expression in co-cultures of TCam-2 and immune cells in the presence or absence of cell–cell contact. Individually cultured cells served as controls. (**A**) IL-2 and IL-6 levels in T cell co-cultures (*n* = 4–5 for IL-2, *n* = 11–13 for IL-6). (**B**) TNFα and IL-6 levels in monocyte co-cultures (*n* = 15). (**C**) CD25 and CD163 expression on monocytes based on frequency or MFI, respectively (*n* = 10–12 for CD25, *n* = 9 for CD163). Exemplary overlay histograms (left panels) and bar diagrams showing the mean ± SEM with individual data points (right panels). Statistical analysis was performed by One-way ANOVA followed by a Tukey’s multiple comparison test (*: *p* < 0.05; **: *p* < 0.01; ***: *p* < 0.001; n.s.: *p* > 0.05).

To address this issue, T cells or monocytes isolated from the peripheral blood of healthy volunteers were co-cultured with TCam-2 cells either directly or separated by a Transwell insert. Neither T cells nor TCam-2 cells produced IL-2 when cultured alone. In the co-cultures, however, IL-2 secretion was induced, an effect which was largely prevented by the presence of a Transwell insert (Figure 1A). Concordantly, the moderately enhanced production of IL-6 in co-cultures of TCam-2 cells with T cells ceased when both cell types were spatially separated (Figure 1A). A similar observation was made for TCam-2 co-cultures with monocytes, where the secretion of large amounts of TNFα and IL-6 was observed upon direct cell–cell contact (Figure 1B). In the presence of a Transwell insert, however, TNFα was hardly detectable and the IL-6 levels were significantly reduced (Figure 1B). While these observations highlight the crucial role of physical contact for immune cell activation, it is noteworthy that in Transwell co-cultures of TCam-2 cells with monocytes, IL-6 secretion was still strongly enhanced (Figure 1B). This finding suggests that soluble factors must contribute to monocyte activation and increased IL-6 secretion by TCam-2 cells too.

To independently confirm the role of cell–cell interaction for immune cell activation by TCam-2 cells, we studied the surface expression of the activation markers CD25 and CD163 on monocytes by flow cytometry. Both proteins were strongly upregulated in the co-cultures compared to monocytes cultured alone, an effect which was largely eliminated in the presence of a Transwell insert (Figure 1C). Collectively, these data suggest that physical contact between TCam-2 and immune cells plays an important although not exclusive role in the ability of seminomatous cells to activate T cells and monocytes and to stimulate the secretion of pro-inflammatory cytokines.

### 2.2. Control of Pro-Inflammatory Gene Expression by TCam-2 Cells

**Figure 2:**
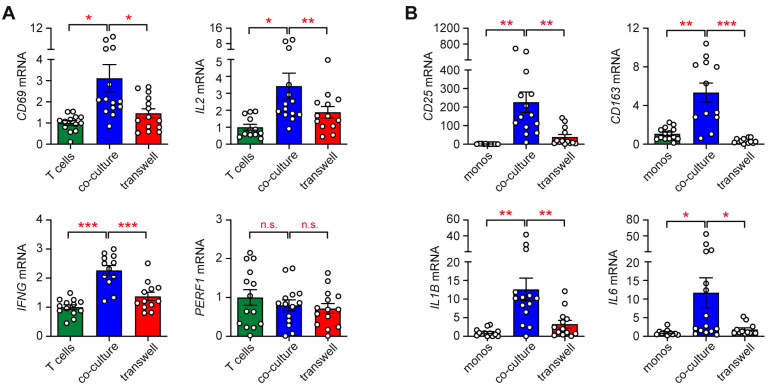
Expression of activation marker and cytokine genes in co-cultures of TCam-2 and immune cells in presence or absence of cell–cell contact. Individually cultured immune cells served as controls. (**A**) *CD69*, *IL2*, *IFNG*, and *PERF1* gene expression in T cells (*n* = 12–15). (**B**) *CD25, CD163, IL1B,* and *IL6* gene expression in monocytes (*n* = 11–14). Bar diagrams showing the mean ± SEM and individual data points. Relative gene expression was calculated by normalization to the housekeeping gene *18SRNA* and arbitrarily set to 1 in immune cells cultured alone. Statistical analysis was performed by One-way ANOVA followed by a Tukey’s multiple comparison test (*: *p* < 0.05; **: *p* < 0.01; ***: *p* < 0.001; n.s.: *p* > 0.05).

Next, we investigated the relevance of direct cell–cell interaction for the regulation of gene expression in immune cell subtypes in TCam-2 co-cultures. In accordance with our previous findings [18], the gene expression of the activation marker *CD69* and the cytokines *IL2* and *IFNG* was significantly upregulated in T cells when co-cultured with TCam-2 cells, while the expression of the effector molecule *PERF1* was unaffected (Figure 2A). Importantly, the observed effect was largely albeit not completely eliminated in T cells when the co-cultures were separated by a Transwell insert (Figure 2A).

To investigate if TCam-2 cells use a similar mechanism to stimulate gene expression in co-cultured monocytes, the activation markers *CD25* and *CD163*, as well as the cytokines *IL1B* and *IL6*, were analyzed. As expected, all genes were strongly induced when cell–cell contact was provided. However, this was not the case when both cell types were spatially separated (Figure 2B). It is noteworthy that *CD25*, *IL1B*, and *IL6* gene expression was still moderately upregulated in Transwell co-cultures compared to monocytes alone despite their spatial separation.

### 2.3. Involvement of IL-6 and TNFα in Immune Cell Activation by TCam-2 Cells

A hallmark of TCam-2 cell co-cultures with T cells or monocytes is their increased secretion of pro-inflammatory cytokines such as IL-6 and TNFα. Hence, we asked whether these cytokines also contribute to the activated phenotype of the immune cells. To address this question, we either neutralized TNFα by adding Adalimumab to the co-cultures or we interfered with the activity of IL-6 by using Tocilizumab, which blocks signaling via its receptor.

**Figure 3:**
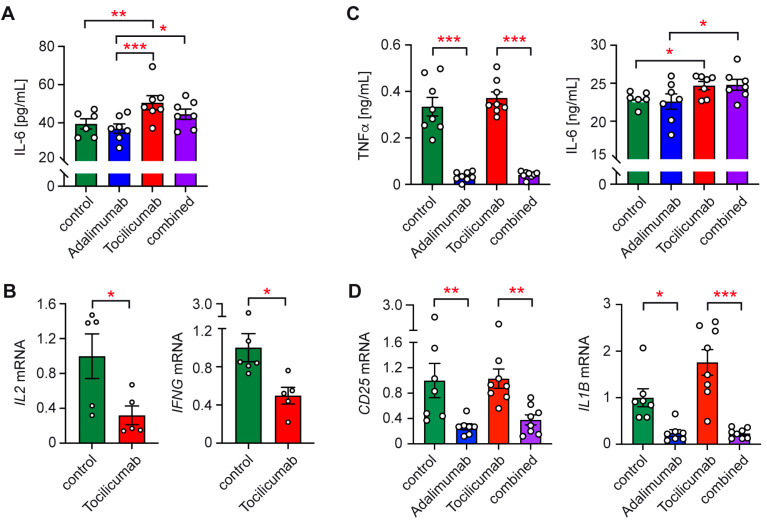
Cytokine secretion and gene expression in co-cultures of TCam-2 and immune cells upon the inhibition of IL-6 or TNFα signaling by monoclonal antibodies. (**A**) IL-6 levels in T cell co-cultures (*n* = 6–7). (**B**) *IL2* and *IFNG* gene expression in T cells from co-cultures (*n* = 4–7). (**C**) TNFα and IL-6 levels in monocyte co-cultures (*n* = 6–8). (**D**) *CD25* and *IL1B* gene expression in monocytes from co-cultures (*n* = 7–8). Relative gene expression was calculated by normalization to the housekeeping gene *18SRNA* and arbitrarily set to 1 for each type of immune cell cultured alone. Statistical analysis was performed by One-way ANOVA followed by a Tukey’s multiple comparison test (*: *p* < 0.05; **: *p* < 0.01; ***: *p* < 0.001).

Initially, we set up co-cultures of TCam-2 and T cells either in the presence of each antibody alone or a combination of both. Our observation that Tocilizumab slightly albeit significantly increased IL-6 levels in co-culture supernatants is indicative of the successful blockade of IL-6 receptor signaling (Figure 3A). In contrast, the neutralization of TNFα with Adalimumab did not have any effect on IL-6 secretion, as expected. Importantly, treatment with Tocilizumab resulted in a reduced gene expression of *IL2* and *IFNG*, indicating that the availability of IL-6 in co-cultures is crucial for T cell activation (Figure 3B).

Subsequently, TCam-2 co-cultures with monocytes were treated with Adalimumab, Tocilizumab, or both antibodies combined. Adalimumab strongly reduced the TNFα levels in the cell culture supernatants, whereas Tocilizumab moderately increased the IL-6 levels, similar to in T cell co-cultures (Figure 3C). These data confirm that both antibody treatments were effective in principle. When we analyzed gene expression, it turned out that Adalimumab reduced *CD25* and *IL1B* mRNA levels regardless of whether it was added alone or combined with Tocilizumab (Figure 3C). In contrast, Tocilizumab had no effect on monocyte gene expression, possibly due to residual IL-6 activity (Figure 3C). We suspect that the lack of detectable effects of Tocilizumab treatment on gene expression in this setup may be due to the large IL-6 concentrations in the monocyte co-cultures, which are approximately 70-fold higher than in the T cell co-cultures and thus presumably overwhelm Tocilizumab’s blocking capacity.

### 2.4. Impact of Conditioned Media on the Subtype Identity of TCam-2 Cells

**Figure 4:**
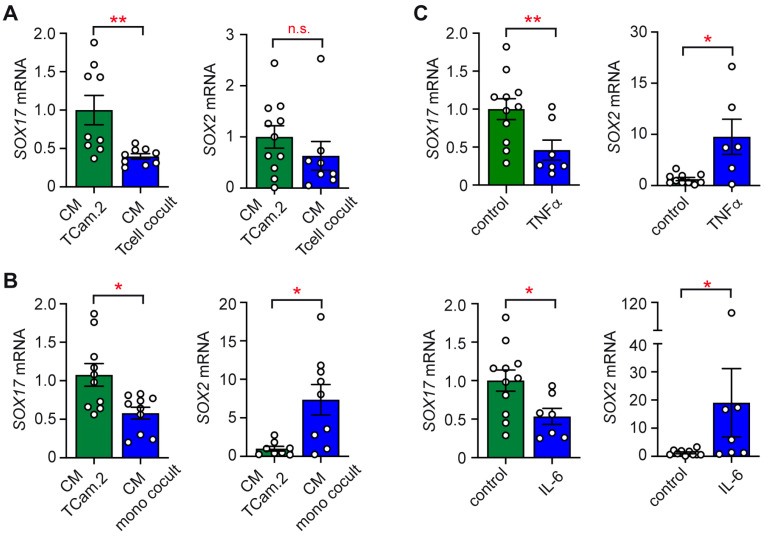
Regulation of gene expression in TCam-2 cells by soluble mediators. (**A**) *SOX17* and *SOX2* gene expression in TCam-2 cells after transfer of conditioned media (CM) from T cell co-cultures or TCam-2 cells alone (*n* = 8–11). (**B**) *SOX17* and *SOX2* gene expression in TCam-2 cells after transfer of CM from monocyte co-cultures or TCam-2 cells alone (*n* = 8–11). (**C**) *SOX17* and *SOX2* gene expression in TCam-2 cells treated with 10 ng/mL of TNFα or IL-6. Untreated TCam-2 cells served as a control (*n* = 6–11). Relative gene expression was calculated by normalization to the housekeeping gene *18SRNA* and arbitrarily set to 1 for each control condition. Statistical analysis was performed by unpaired *t*-test (*: *p* < 0.05; **: *p* < 0.01; n.s.: *p* > 0.05).

Having established that co-culturing TCam-2 cells with immune cells elicits the secretion of pro-inflammatory cytokines, we aimed to ascertain whether soluble factors reciprocally influence the stemness features of seminomatous cells. To address this question, TCam-2 cells were cultured alone or co-cultured with T cells or monocytes for 24 h. Then, conditioned media (CM) from each of them were transferred to fresh TCam-2 cell cultures, and another 24 h later, an RT-qPCR analysis was performed. The CM from the T cell co-cultures reduced *SOX17* expression compared to the CM from TCam-2 cells cultured alone but had no impact on *SOX2* (Figure 4A). Similarly, the CM collected from the monocyte co-cultures diminished *SOX17* expression but concomitantly upregulated *SOX2* (Figure 4B). We conclude that soluble mediators released by immune cells promote a dynamic and complex shift to a more dedifferentiated TGCT phenotype.

### 2.5. Phenotypic Switch of TCam-2 Cells in Response to Cytokines

Considering that high amounts of TNFα and IL-6 were produced during the co-cultures, we tested the genuine influence of these cytokines on the phenotype of the seminomatous cells. To this end, we incubated TCam-2 cells for 24 h with 10 ng/mL of recombinant human TNFα or IL-6. The RT-qPCR analysis confirmed that the *SOX17* mRNA levels in the TCam-2 cells were diminished in response to both cytokines. Concurrently, the expression of *SOX2* was enhanced, although the observed effect was highly variable (Figure 4C). This possibly relates to the heterogeneity of TCam-2 cells or the delayed regulation of *SOX2* expression compared to *SOX17*. Future studies should therefore address additional dedifferentiation markers like *NANOG* and include longer durations of co-cultures. Collectively, our data suggest that pro-inflammatory cytokine release by immune cells could promote the dedifferentiation of seminomatous cells in the TME.

## 3. Discussion

Seminomas, as an important example of a type II TGCT, represent an immunological tumor with relatively high numbers of infiltrating immune cells and a predominantly pro-inflammatory TME signature [10,12,31,32]. In an effort to understand the mechanistic basis of the tumor–immune interactions within the TME, we used several approaches based on our established in vitro co-culture model using the seminomatous cell line TCam-2 and purified immune cell subsets from healthy human blood donors. In particular, we aimed to further characterize the communication between immune and tumor cells via physical cell–cell contact and soluble mediators. To address this issue, we used a simplistic model of the TME based on co-culturing TCam-2 cells with individual, highly purified immune cell subtypes. While this approach allows the observed effects to be clearly attributed their cellular source, it has the disadvantage that it lacks stromal components such as cancer-associated fibroblasts and other immune cell subtypes present in the TME of seminomas in situ. Thus, future studies using organoids or patient-derived xenografts could further elucidate their contribution to tumor–immune interactions.

T cells and monocytes secrete large amounts of pro-inflammatory cytokines, such as IL-2, IL-6, and TNFα, when co-cultured with TCam-2 cells, an effect which is significantly reduced when immune and tumor cells are separated by a Transwell insert. This finding indicates that a direct interaction between both cell types is indispensable for a robust cytokine response. However, our data also revealed that even in the absence of physical cell–cell contact, meaningful amounts of cytokines were still released, particularly IL-6 in co-cultures of monocytes with TCam-2 cells. This finding suggests that soluble factors such as CXCL10 may also contribute to monocyte activation by TCam-2 cells, warranting further investigation to identify the nature of these mediators, for instance, through the cytokine profiling of co-culture supernatants [16]. Taken together, TCam-2 cells communicate with T cells and monocytes via both cell–cell contact and soluble mediators, although to a different degree.

Our flow cytometric and gene expression data also highlight the important albeit not exclusive role of cell–cell contact for immune cell activation by TCam-2 cells. That being said, the increased CD25 and CD163 surface expression on monocytes in the co-cultures was inhibited in the presence of Transwell inserts. In accordance with these results, the enhanced gene expression of activation markers (*CD69*, *CD25*, *CD163*) and cytokines (*IL2*, *IFNG*, *IL1B*, *IL6*) by T cells and monocytes in the co-cultures with TCam-2 cells strongly depended on direct interactions between each cell type. Nonetheless, a moderate increase in gene expression was also noted in the Transwell co-cultures compared to immune cells cultured alone. Hence, the engagement of receptors by membrane-bound ligands, which results in the initiation of intracellular signaling cascades necessary for transcriptional activation, plays a crucial role in immune cell activation by seminomatous cells, but soluble factors, either secreted by tumor or immune cells, contribute to this effect too.

In the majority of cancer entities, tumor cells exert a pronounced inhibitory effect on infiltrating immune cells via various membrane-bound checkpoint molecules like PD-L1 or CD24 [33,34,35]. In addition, immunosuppressive cytokines such as IL-10 and TGFβ are frequently detected in the TME. In TGCTs, especially in seminomas, the situation is different since these tumors are characterized by a mostly pro-inflammatory TME. However, the specific ligand–receptor pairs that mediate the cell–cell contact between seminomatous cells and immune cells and thereby contribute to their activation in vitro and in vivo are unknown. Candidates possibly involved in modulating T cell activity include CD28/CD80, ICOS/ICOSL, OX-40/OX-40L, and B7-H6/NKp30, but very little is known as to their role in tumor–immune communication [13]. While most of these signaling pathways result in T cell inhibition, a few of them have also been confirmed to have stimulatory effects. ICOSL is induced in tumor cells and promotes CD8 T cell cytotoxicity [36]. B7-H6 is expressed by several malignant tumors and interacts with NKp30 in NK and CD8 T cells, thereby inducing cytotoxicity as well [37]. Moreover, a low expression of CD80 resulting in T cell co-stimulation has been detected in several tumor entities, such as colon carcinoma [38] and melanoma [39]. These examples warrant further investigation to identify relevant ligand–receptor pairs in the TME of seminomas and thereby aid in the identification of new drug targets.

Macrophages can commit to different phenotypes that are either characterized by pro-inflammatory or anti-inflammatory features. The enhanced surface expression of CD25 and CD163 in monocytes in the TCam-2 cocultures in combination with the increased TNFα and IL-6 production indicates a strong commitment towards an M1-like phenotype. Such polarization of monocytes and macrophages is generally associated with improved survival and a favorable clinical outcome in various types of cancers [40]. Namely, they produce reactive oxygen species and pro-inflammatory cytokines and can inhibit cancer growth, induce apoptosis, and reinforce phagocytosis. M1-polarization typically occurs in response to microbial molecules such as LPS. TLR4 engagement is particularly important in this process and can even cause repolarization of tumor-associated M2-like cells [41]. While microbial products do not play any role in tumor–immune interactions, a variety of molecules have been described that can mimic the effects of LPS by binding either to TLR4 or its coreceptor MD-2 and thereby promoting the activation of monocytes and macrophages with a pro-inflammatory phenotype [42]. While such a mechanism has mostly been studied in the context of allergies, it is very conceivable that it could be involved in shaping the TME too. However, to the best of our knowledge, the expression of potential TLR ligands in TGCTs has not been studied so far.

While T cell and monocyte activation was strongly reduced in the Transwell co-cultures with TCam-2 cells, we still noted a moderately enhanced expression of activation markers and cytokines compared to immune cells cultured alone. Similarly, cytokine secretion was partially induced in the absence of direct cell–cell contact. This observation relates to IL-2 secretion by T cells, IL-6 secretion by monocytes, *IL2* and *IFNG* expression by T cells, and both CD25 surface and gene expression by monocytes. Hence, soluble factors apparently contribute to the activation of immune cells by TCam-2 cells too. A candidate that could possibly account for this effect is IL-6, which is secreted by TCam-2 cells in meaningful amounts. Evidence in support of this notion comes from our observation that blocking the IL-6 receptor with Tocilizumab reduced *IL2* and *IFNG* gene expression by T cells. It is known that IL-6 activates T cells via STAT3 signaling, promotes the development of effector cells, and counteracts the generation of regulatory T cells [43]. It is thus plausible that its release by TCam-2 cells stimulates T cells to some extent even in the absence of cell–cell contact, which is prevented if IL-6 receptor signaling is blocked by Tocilizumab. In the case of monocytes and macrophages, IL-6 has been shown to induce an M1-like phenotype and could thus contribute to monocyte activation in the TCam-2 co-cultures [44]. However, Tocilizumab failed to influence monocyte gene expression in our experiments, which can possibly be explained by the large amounts of IL-6 released under co-culture conditions, hampering the complete blockade of its signaling capacity. In contrast, monocyte activation was diminished by the neutralization of TNFα. Since TCam-2 cells do not produce this cytokine, we postulate that it must be secreted by monocytes and promotes their own activation in an autocrine manner, an effect which is inhibited by treatment with Adalimumab. Taken together, we unveiled the functionally discrete roles of IL-6 and TNFα in shaping the pro-inflammatory TME in seminomas, although we cannot exclude the possibility that tumor cells produce additional soluble factors like the chemokines CXCL10 that are involved in this process.

While Tocilizumab and Adalimumab have shown promising results in modulating the TME in vitro, challenges such as systemic toxicity or compensatory cytokine signaling must be solved in interventional studies in a clinical setting. Adalimumab has not been tested for cancer therapy so far, and trials with Tocilizumab are still in an early phase [45]. Moreover, neither of these antibodies has been tested in the context of TGCTs so far. The administration of recombinant TNFα shows powerful anti-tumor activity in vivo, but early-stage clinical trials were hampered by severe toxicities [46]. Treatment with recombinant IL-6 showed only minimal responses in tumor therapy, accompanied by marked adverse effects [47]. Whether such an interventional strategy could be a promising approach in TGCT therapy, considering their exceptionally pro-inflammatory TME, must be addressed in future clinical trials.

Regarding previously published work attesting the central roles of IL-6 and TNFα in TGCTs as well as our own results implicating these cytokines in tumor–immune interactions [18,25,28,29,48], we wondered about their specific relevance for the effect of the cytokine milieu on the tumor cells themselves. Conditioned media from co-cultures of T cells or monocytes with TCam-2 cells caused partial dedifferentiation of the latter. *SOX17*, a marker of seminomatous identity, was consistently reduced, while *SOX2*, which is associated with pluripotency and embryonal carcinoma-like features, was upregulated or unchanged. The treatment of TCam-2 cells with IL-6 or TNFα mimicked these effects, suggesting that these pro-inflammatory cytokines account for the downregulation of *SOX17* and the trend of concomitant *SOX2* upregulation in TCam-2 cells in co-cultures. Importantly, the observed effects on *SOX17* were robust throughout the different assays, whereas the regulation of *SOX2* expression was more variable. Overall, the present work confirms previously published results from in vitro studies with TGCT cell lines [18,25]. Moreover, it aligns with studies showing that pro-inflammatory cytokines can induce transcriptional and epigenetic reprogramming in tumor cells, thereby contributing to lineage plasticity [49,50,51]. As a matter of fact, such plasticity has previously been implicated in relapse and chemoresistance in TGCTs, especially if the tumors acquire non-seminomatous features during progression [19]. Collectively, the observed shift in seminomatous cells toward a dedifferentiated phenotype, marked by reduced *SOX17* and variable *SOX2* upregulation, may contribute to increased malignancy or chemoresistance in TGCT patients [19]. It is well known that the dedifferentiation of TGCTs is associated with altered *OCT3/4* expression and DNA methylation, as well as changes in the abundance of pro-apoptotic factors, which influence sensitivity to cisplatin-based therapy and thus clinical outcomes [52,53]. Hence, our findings suggest that the cytokine-driven initiation of a dedifferentiation program could be a promising therapeutic target in TGCTs.

## 4. Materials and Methods

### 4.1. Leukocyte Isolation

Buffy coats were prepared from blood donations from anonymous healthy volunteers. PBMCs were isolated by density gradient centrifugation, as described previously [18]. The sorting of individual leukocyte subpopulations was achieved with the EasySep^TM^ human T cell or Monocyte Isolation Kit (Stemcell Technologies, Cologne, Germany) according to the instructions of the manufacturer. The purity of cell preparations was >95% in the case of T cells and >85% in the case of monocytes, as determined by flow cytometric analysis. Although the presence of minor contaminants, especially in monocyte preparations, could influence cytokine secretion and gene expression, their impact is likely minimal given the overall high purity achieved by magnetic cell sorting.

### 4.2. Cell Culture

The human cell line TCam-2, which is derived from a testicular seminomatous germ cell tumor, was cultured in DMEM/Ham’s F-12 L-Glutamine medium (Capricorn Scientific, Ebsdorfergrund, Germany) supplemented with 10% FCS and 1% penicillin/streptomycin (ThermoFisher, Waltham, MA, USA) at 37 °C and 5% CO_2_. To set up co-cultures with leukocyte subpopulations, TCam-2 cells were seeded at a concentration of 7.5 × 10^5^/mL in a volume of 2 mL in 6-well plates and incubated for 24 h. On the next day, T cells (1.5 × 10^6^ cells/mL) or monocytes (1 × 10^6^ cells/mL) were added in a similar volume and co-cultured for another 24 h. As a control, immune cells and TCam-2 cells were cultured alone. To assess the relevance of cell–cell contact, Transwell inserts with a pore size of 0.4 µm (ThermoFisher) were used to separate both cell types in 24-well plates, allowing for the diffusion of soluble mediators while preventing cell migration. Specifically, TCam-2 cells were seeded at a concentration of 7.5 × 10^5^/mL in a volume of 500 µL in the lower chamber and cultured for 24 h. T cells or monocytes, also resuspended in a volume of 500 µL, were subsequently placed in the upper chamber in the same concentrations as indicated above and co-cultured for another 24 h. Adalimumab and Tocilizumab (Hycultec, Beutelsbach, Germany) were added at a final concentration of 10 µg/mL, whereas recombinant human TNFα and IL-6 (Peprotech, Osterode, Germany) were added at a final concentration of 10 ng/mL. TCam-2 cells were collected by trypsinization according to standard protocols. The duration of the co-culture experiments was chosen on the basis of preliminary experiments indicating that strong immune cell activation was achieved after 24 h at the mRNA and protein level while optimal vitality for all cell types was still retained.

### 4.3. Quantitative RT-PCR (RT-qPCR)

The Quick-RNA MiniPrep kit (Zymo Research, Irvine, CA, USA) was used to isolate total RNA from immune or TCam-2 cells, which was reverse transcribed into cDNA with the iScript kit (Bio-Rad, Munich, Germany). RT-qPCR analysis was performed on an ABI 7500 instrument (ThermoFisher) utilizing SYBR Green Master Mix from the same company. Relative gene expression was calculated with the ΔΔCt method by normalization to the housekeeping gene *RNA18S*. Primers were synthesized by Metabion (Planegg, Germany); their sequences have been published previously [18].

### 4.4. Flow Cytometry (FACS)

Leukocytes were incubated with fluorochrome-conjugated monoclonal antibodies according to standard protocols. Antibodies were purchased from BioLegend (Uithoorn, The Netherlands; clone names in brackets): anti-hHLA-DR (I.243), anti-hCD25 (BC96), anti-hCD14 (HCD14), and anti-hCD163 (GHI/61). Data were recorded using a FACS Canto II device (BD Bioscience, Heidelberg, Germany) and analyzed with the help of FlowJo^®^ software (v10.7.0; Tree Star, Ashland, OR, USA). Monocytes were identified by initially gating the recorded events on the basis of their forward and side scatter and subsequently defined as HLA-DR^+^ CD14^+^ cells. The regulation of CD25 expression was assessed by analyzing the percentage of CD25^+^ cells, while CD163 regulation was determined on the basis of the cells’ mean fluorescence intensity (MFI).

### 4.5. Enzyme-Linkened Immunosorbent Assay (ELISA)

Commercially available ELISA kits were employed to determine the concentrations of IL-2, TNFα, and IL-6 (BioLegend) in appropriately diluted cell culture supernatants following the instructions of the manufacturer. Absorption was measured using a BioTek Power wave 340 Plate Reader (BioTek Instruments, Wetzlar, Germany).

### 4.6. Statistical Analysis

One-way ANOVA was used to compare several experimental groups, followed by Tukey’s test to control for multiple comparison, ensuring robust statistical analysis. In the case of two experimental groups, an unpaired *t*-test was used. GraphPad Prism^®^ software (v5.04; San Diego, CA, USA) was employed in all statistical analyses. Data are depicted as bar diagrams showing the mean ± SEM and individual data points as open circles. Levels of significance: *: *p* < 0.05; **: *p* < 0.01; ***: *p* < 0.001; n.s.: *p* > 0.05.

## 5. Conclusions

Our mechanistic studies on seminoma–immune interactions provide evidence that direct cell–cell contact, in particular, but also the secretion of soluble factors such as IL-6 and TNFα shape the pro-inflammatory micromilieu in our in vitro co-culture model and presumably also in the TME of TGCT patients. More precisely, we propose that TCam-2 cells express membrane-bound stimulatory ligands that bind to receptors present on the surface of T cells and monocytes and thereby induce their activation. IL-6 and possibly other mediators released by TCam-2 cells reinforce these signals, although their functional potency is limited in the absence of physical interactions. Irrespectively, cytokines additionally fulfill a role in reciprocal signaling by inducing the dedifferentiation of seminomatous cells. Taken together, tumor and immune cells in the TME of TGCTs communicate in a complex manner using different mechanisms. Although our study exclusively relies on a single cell line, which may not fully capture the heterogeneity of seminomas and thus requires validation in primary tumor specimens, we are convinced that our findings warrant a search for new therapeutic targets to selectively interfere with cellular communication in the TME of TGCT patients.

## Data Availability

Data and material are available upon reasonable request.
